# A Review of Different Aspects of Applying Asphalt and Bituminous Mixes under a Railway Track

**DOI:** 10.3390/ma14010169

**Published:** 2020-12-31

**Authors:** Kazem Jadidi, Morteza Esmaeili, Mehdi Kalantari, Mehdi Khalili, Moses Karakouzian

**Affiliations:** 1 Howard R. Hughes College of Engineering, University of Nevada Las Vegas, Las Vegas, NV 89154, USA; Mkar@unlv.nevada.edu; 2 School of Railway Engineering, Iran University of Science and Technology, Tehran 13114-16846, Iran; m_esmaeili@iust.ac.ir; 3 Department of Civil Engineering, Roads Research Institute, D-57068 Siegen, Germany; kalantari@bau.uni-siegen.de; 4 Geosyntec Consultants, Johnson City, TN 37601, USA; MKhalili@geosyntec.com

**Keywords:** railway, slab track, asphalt, bitumen, underlay, overlay, asphalt concrete, mortar

## Abstract

Asphalt is a common material that is used extensively for roadways. Furthermore, bituminous mixes have been used in railways, both as asphalt and as mortar. Different agencies and research institutes have investigated and suggested various applications. These studies indicate the benefits of bituminous material under railways, such as improving a substructure’s stiffness and bearing capacity; enhancing its dynamic characteristics and response, especially under high-speed train loads; waterproofing the subgrade; protecting the top layers against fine contamination. These potential applications can improve the overall track structure performance and lead to minimizing settlement under heavy loads. They can also guarantee an appropriate response under high-speed loads, especially in comparison to a rigid slab track. This review paper documents the literature related to the utilization of asphalt and bituminous mixes in railway tracks. This paper presents a critical review of the research in the application of asphalt and bituminous mixes in railway tracks. Additionally, this paper reviews the design and construction recommendations and procedures for asphalt and bituminous mixes in railway tracks as practiced in different countries. This paper also provides case studies of projects where asphalt and bituminous mixes have been utilized in railway tracks. It is anticipated that this review paper will facilitate (1) the exchange of ideas and innovations in the area of the design and construction of railway tracks and (2) the development of unified standards for the design and construction of railway tracks with asphalt and bituminous mixtures.


**Contents**
1.Introduction 22.Asphalt Pavement Applications in Railway Track Support Systems 3
*2.1.*

*Asphalt in Ballasted Railway Tracks*
 4
*2.2.*

*Asphalt in Ballastless Tracks*
 4
*2.3.*

*Asphalt Mortar in Ballastless Track*
 63.Recommendations on the Mix Design of Asphalt for Railway Tracks 7
*3.1.*

*Volumetric Properties*
 7
*3.2.*

*Aggregate Gradation*
 94.Structural Properties of Asphalt Layer 105.Asphalt Performance and Constructability 126.Review of Case Studies 12
*6.1.*

*Ballasted Railway Tracks with Asphalt*
 12
*6.2.*

*Ballastless Railway Track with Asphalt*
 13
*6.3.*

*Railway Tracks with Modified Asphalt Mixtures*
 14
*6.4.*

*Railway Tracks with Asphalt Mortar*
 147.Asphalt Application in Urban Railway Tracks 158.Summary and Conclusions 15References
 16

## 1. Introduction

In traditional railway systems, the ballast and sub-ballast layers are considered the main elements of the track for providing the strength required to support the track structure and protect the subgrade. Recent efforts include adding or replacing some of these layers with a concrete or asphalt layer. Concrete, which is mostly used in ballastless tracks, has been studied and developed extensively; however, the use of an asphalt layer needs more focus. During this literature review, the authors noticed that, while asphalt is among the common materials used in railway construction in many countries, a review of its use has not been gathered in a single paper. It is necessary to collect the available documents and research results in a comprehensive literature review and highlight the important findings of these investigations.

The application of bitumen as a pavement material dates back to the 1800s when a sheet of asphalt layer was placed under a concrete base. Until the 1930s, the structural design and construction of pavements were based only on experience. However, after this date, early efforts to review the structural design of flexible pavements began [1]. The first applications of asphalt as a track bed date back to the 1960s and 1970s. Despite the rise in demand for slab track construction, the use of asphalt in railways did not grow as extensively as concrete. However, considering the fact that the lifetime of asphalt pavement can be extended to over 50 years [2], the total cost for its use could be less than a traditional ballasted track.

Since the 1970s, different applications of asphalt have been introduced and used in various places. In the United States, asphalt application in railways started in the early 1980s. Early applications mainly aimed at improving the strength of the track structure, specifically during renovation projects on existing lines. In general, the designs included adding a 15 cm layer of hot mix asphalt concrete between the ballast and the subgrade. Based on the literature, almost the same design application and configuration are still being used today [3]. Efforts are in place to design better mixes for all sorts of pavements, including highways, airfields, and railway layers, as well as improving construction techniques and production methods [4].

Many other countries have also been using asphalt products in railway structures. For instance, Japan, Spain, and Italy are among the countries that have successfully applied asphalt in their track systems [5]. In Germany, a method called Getrac, which is a system with an asphalt base layer, was approved as a construction type of ballastless track system for high speeds. Studies indicated that the system has benefits similar to other ballastless types while being fast and easy for construction. Asphaltic material has the advantage of higher flexibility compared to concrete, which makes it an ideal material to design and build a flexible system with a good bearing capacity for high-speed lines [6].

Table 1 presents the estimated lengths of railway tracks in several countries with different types of bituminous layers. The asphalt layer thicknesses vary for ballasted and ballastless systems; the overall thickness that is the most common in each country is presented.

It is important to mention that, in the beginning, there were several problems involved with asphalt construction technology, especially fixing the track panel on a material with viscous behavior. This led to the introduction of different sleeper types and fastening systems. Through new developments in paving machinery and construction technology, it was possible to easily lay asphaltic layers, and at the same time, more precisely, which could satisfy the required installation tolerances [13,14].

In terms of high-speed railway construction methods, the asphaltic track system is now one of the accepted standard systems in the German railway [15]. Compared to hydraulically bonded base layers (cement-stabilized or concrete materials), they can be loaded shortly after the end of compaction. This great advantage of asphalt makes it a favorable material for bearing capacity improvements in the upgrading of existing tracks for higher speeds, or the rehabilitation of existing high-speed ballasted tracks. To satisfy the stiffness and dynamic requirements of the load-bearing system, hot mix asphalt has been shown to be appropriate material. It is necessary to mention that in order to satisfy the lifecycle of the asphalt layer, fatigue damage and settlement are among the main aspects of the design criteria for this layer. Both of these aspects are related to the number of train passes. In this regard, Société nationale des chemins de fer français (SNCF) recommends the following mechanical properties for the asphalt layer under a high-speed railway track [5]:

- stiffness modulus ≥ 11,000 MPa (at 15 °C and 10 Hz)
- fatigue ≥ 100 microstrain.

The European Union’s strategy for after 2030 is to build maintenance-free tracks in tunnels and other appropriate places [16], which means that there will be opportunities for asphalt and other bituminous materials as alternatives for concrete and cemented products in ballastless track systems. In addition to the European Union, investigators in Australia have been working on new methods for the construction of a high-speed railway with asphalt or asphaltic mortar [17].

The main applications of bituminous materials in a railway track bed include an underlayment between the ballast and sub-ballast layers, a replacement for the sub-ballast, a layer between the concrete slab or sleeper panels and the subgrade, a structural layer of an urban railway, a bituminous mortar layer between the concrete slab and the subgrade, a waterproofing layer, and a bearing capacity layer during the upgrade of existing lines for higher speeds. Considering the inherent characteristics of hot mix asphalt, and asphaltic materials over concrete and granular material in general, they have a high potential of being applied in existing railway tracks or as an element in the design of new railway lines. The next sections will cover various applications of asphalt and bituminous materials under railway tracks, along with technical recommendations, as well as the results of case studies on asphalt-based track systems.

## 2. Asphalt Pavement Applications in Railway Track Support Systems

Various asphalt types and bituminous products have been introduced and used in railway construction. These include the applications of hot mix asphalt (HMA), warm mix asphalt (WMA), mastic asphalt, and asphalt mortar, with various types of bitumen, from normal bitumen and emulsion bitumen up to rubber and polymer-modified ones. The main reason behind most of these applications is to decrease the level of stress and strain transferred to the subgrade. Additional studies have looked at the waterproofing properties of asphaltic materials, which is also a form of protection of the subgrade.

In general, asphalt applications under railway tracks can be divided into three major categories: the first uses an asphalt mixture with a ballasted track, the second uses asphalt directly under railway tracks, and the third uses asphalt mortar.

### 2.1. Asphalt in Ballasted Railway Tracks

**Figure 1:**
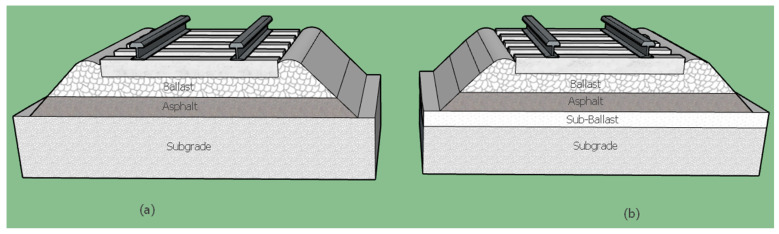
Schematic of combinations of asphalt layers in a ballasted track bed: (**a**) asphalt as a sub-ballast and (**b**) asphalt between the ballast and the sub-ballast.

One of the main applications of asphalt mixtures is with a ballasted track. Figure 1 shows a schematic of a ballasted railway track with asphalt pavement as a supporting component. This method, which is common in the United States, is called “asphalt underlayment,” and is composed of a layer of asphalt that is used instead of sub-ballast, or between the sub-ballast and ballast layers. In this system, the asphalt layer generally extends around 30 cm beyond the ends of the crossties on both sides.

The benefits of asphalt underlayment are higher track stiffness and bearing capacity, improved track geometrical stability, decreased vertical track deformation, waterproofing, enhanced durability, and reduced vibration [13]. Asphalt underlayment is recommended for at-grade rail crossings in order to minimize the track and road settlement in the crossing area over time [18]. Caltrain in San Francisco and Metrolink in Los Angeles were among the first urban railways to utilize a layer of asphalt with 20 cm and 15 cm of thickness under crossovers and crossings, respectively. Additionally, most new crossovers and turnouts on both lines are built over an asphalt layer [19]. The thickness of the ballast-covering layer has been shown to have a noticeable influence on the amount of maximum stress and strain produced at the bottom of the asphalt layer. The component properties of a typical railway track with asphalt underlayment used in the United States are presented in Table 2 [20,21].

### 2.2. Asphalt in Ballastless Tracks

**Figure 2:**
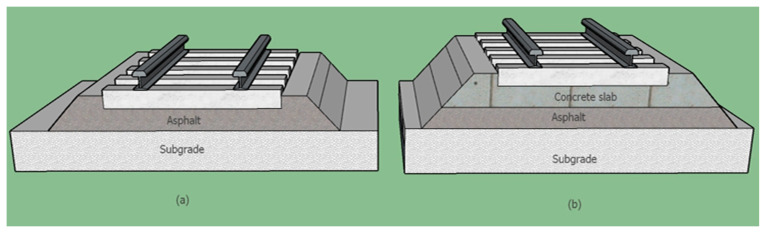
Schematic of combinations of ballastless layers in a track bed: (**a**) asphalt slab track and (**b**) asphalt between concrete slab and subgrade.

The second category uses asphalt in ballastless tracks. In this method, asphalt is placed directly under the sleepers (crossties) or a concrete slab. This method acts similarly to a slab track. European countries first introduced this method specifically for constructing high-speed railways and urban transit lines. Figure 2 shows a schematic structure of a ballastless railway track with an asphalt layer.

In the United States, asphalt is generally used under a concrete slab track. It is limited to lines with heavy freight traffic and for maintenance purposes. For the Long Island Station (LIRR), constructed in the 1980s, a layer of 10 to 15 cm of asphalt-treated sub-base was laid over a compacted subgrade to support 30 to 35 cm concrete slab track panels. It is notable that the slab track performance has not diminished, despite having been in service for a long time [22,23]. European countries have a higher tendency to use asphalt for ballastless tracks. Table 3 presents the specifications of various ballastless systems that include an asphalt base used on European railways.

**Figure 3:**
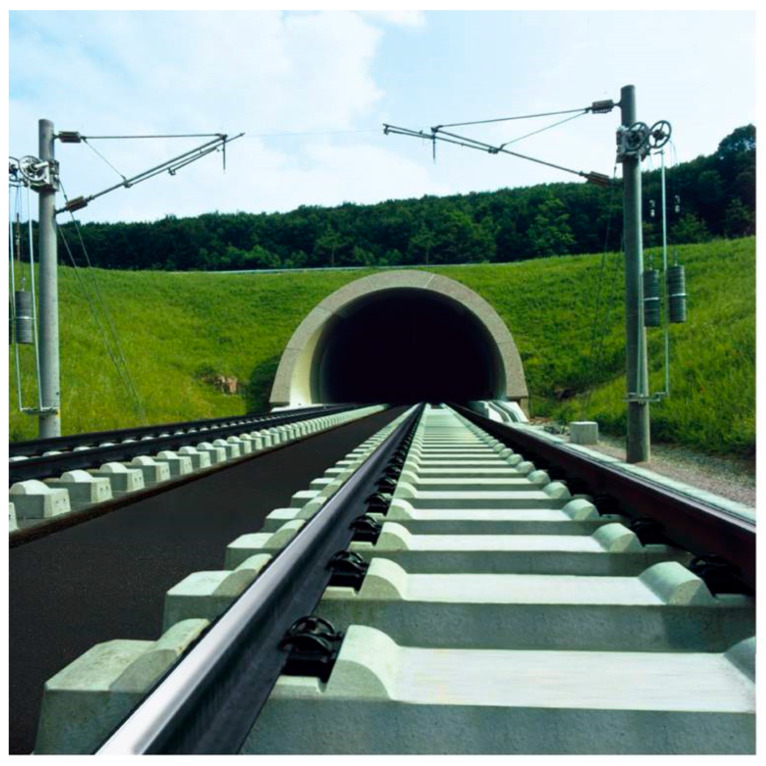
Getrac ballastless system with an asphalt layer (with permission from RAIL.ONE) [27].

An ATD (asphalt track bed directly laid on the sleeper) system [24] includes an asphalt layer over a cement-stabilized sub-base, and the sleepers sit on top of this asphalt layer. Studiengesellschaft AsphalT Oberbra (SATO) and Feste Fahrbahn Y-Sleeper (FFYS) systems include Y-shape sleepers anchored into the asphalt layers under them. For the Walter method, monoblock sleepers are anchored to the asphalt layer [2] Getrac, which is the name of a ballastless slab track system developed in Germany, includes two sub-systems: Getrac A1 and Getrac A3. The main difference between them is related to the sleeper width. Getrac sleepers are quite heavy and have an anchor block to maintain the lateral stability of the system. The precision degree for asphalt base slab tracks is ±2 mm in slab height [25,26,27]. Figure 3 demonstrates two sections of a ballastless Getrac track system. It is important to mention that, in some sections, in order to provide lateral resistance, a layer of ballast is added at the end of the sleepers, on the track shoulders. The ballast in these sections is placed over the track shoulders after constructing the railway slab track.

### 2.3. Asphalt Mortar in Ballastless Track

**Figure 4:**
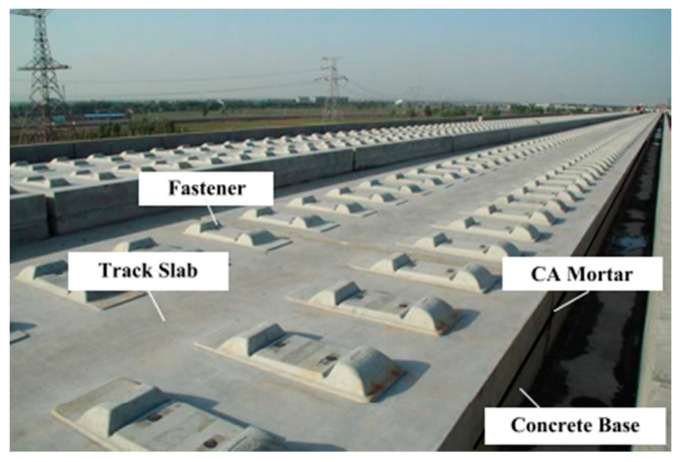
Cement Asphalt (CA) mortar under a slab track (with permission from American Society of Civil Engineers (ASCE)) [33].

In Japan, China, and Italy, to protect the subgrade, high-speed railway tracks are constructed over a layer of 5 to 8 cm of asphalt mortar. The design method used in Japan includes a prefabricated concrete slab over a bitumen-cement mortar [28,29]. In this method, the bitumen-cement mortar is one of the key components of the track structure. The asphalt layer transfers the load and distributes it uniformly on the subgrade. It also creates a waterproof surface, which keeps the subgrade moisture content constant. In addition, a higher mortar stiffness leads to a slight decrease in concrete slab displacement based on a dynamic analysis of the slab track behavior, although this influence could be neglected [30]. A study on a Chinese high-speed railway suggested a design with a thicker slab, lower rail fastening stiffness, and higher mortar stiffness to decrease the slab bending stresses [31]. Chinese high-speed railway designers consider mortar as a semi-rigid composite, which is injected between the track slab and the concrete bed. Mortar’s mechanical properties, elastic modulus, and durability are dominant factors in determining the safety and service life of a high-speed railway [32]. It is important to mention that extreme climate has a significant influence on mortar’s performance [33]. Figure 4 presents the bituminous mortar (bitumen-cement mortar) layer under a slab track.

## 3. Recommendations on the Mix Design of Asphalt for Railway Tracks

An important concern for the use of asphalt on a railway track is the mix design issue. Looking at the literature, different agencies and researchers have used various volumetric properties, aggregate types, and gradations. The following two sections present the various volumetric properties of asphalt mixtures and aggregate gradation.

### 3.1. Volumetric Properties

In order to produce asphalt, European countries have used their own national procedures, while the USA and many other countries have relied on the Marshall mix design and properties. Despite the fact that the Marshall design is a relatively old method, it has been proven to be appropriate for both the underlayment and overlayment methods. Recent studies have been based on applying Superpave recommendations for designing asphalt mixtures for railways. The specifications of the American Society for Testing and Materials (ASTM) D-3515 are used to produce asphalt mixtures for railroad structures in the USA [20]. A typical mixture design based on the Marshall method is presented in Table 4.

ESALs are the equivalent single axle loads, with the most common equivalent load in the USA being 18,000 lb (80 kN). In the Marshall mix design method, the compaction (blow number) is generally 35, 50, or 75 strikes on each side of the cylindrical sample; stability is the maximum load capacity of the specimen; flow refers to the specimen’s deformation, which is measured in 0.25 mm; air voids are the total volume of air voids in the compacted specimen [34].

The recommendations of this table were prepared based on highway loading criteria. For railway applications, it is crucial to optimize the mixture design in order to achieve a balance between the resistance to permanent deformation and fatigue cracking [35]. The authors could not find mix design recommendations for all European countries, but as an example, in an investigation carried out in Italy, asphalt samples were made based on the Italian Standard in the experimental work [36]. Table 5 presents some of these specifications gathered from different studies. As indicated in this table, most studies have used methods similar to the Marshall mix method to design their asphalt mixtures. It has to be added that the reason for some cells being empty in Table 5 and Table 6 is because the authors did not find the data relevant to these parameters in the given references. One reason for this could that many countries have developed their own standards, where these national standards are usually in languages other than English. For instance, in France, the SNCF has used the French asphalt type GB4 in developing asphalt layers under high-speed railways, in which the asphalt is compacted with a gyratory base method [37].

Moreover, the authors did not find any specific analysis within these documents about the comparison between different mix designs or aggregate gradations. However, the authors believe it is important for those who read this article to know the mix design properties and gradations being used by some studies. It may help some researchers to define a project in order to discover the influence of these parameters on asphalt under railways.

In this table, V_a_ is the air void ratio, VFA represents the voids filled with asphalt, and VMA represents the voids in mineral aggregates. Some of these specifications are based on European standards and specifications; to design asphalt mixtures for tracks inside tunnels, manufactured asphalt must be easy to handle. Using warm mix asphalt allows for decreasing the compaction temperature by around 30 °C compared to conventional HMA [45]. In order to provide a waterproof coating, especially for high-speed railway tracks, mastic asphalt is a good and suggested option, especially in cold areas, to minimize the damage caused by freeze and thaw [46].

### 3.2. Aggregate Gradation

The aggregate gradation that has been used to produce asphalt mixtures for some case studies are presented in Table 6. One study recommended keeping the aggregate’s maximum size under 25 mm and mixing it with approximately 5.8% of binder [47]. Other than that, the authors did not find any technical properties or differences that were specifically related to a recommended gradation for these types of asphalt mixes. In most studies, the researchers used the gradation that is typically used in highways. Moreover, Spanish railway authorities provided the following recommendations to cover the mechanical characteristics of the aggregate for asphalt layer under a railway [5].

- sand equivalent >50;
- angular particles >90%;
- Los Angeles Abrasion Test >25%;
- Elongation and Flakiness Indexes <25;
- filler >50%.

**Table 6:** Aggregate gradation for asphalt mixtures on railways.

Sieve Size/Number	Passing Percentage (%)					
	[5]	[23]	[48]	[49]	[35]	[42]
37.5 mm				100		100
31.5 mm	100	100	100	-		90–100
25.4 mm	-	90–100	-	90–100		-
22.4 mm	90–100	-	92.39	-		-
19 mm	-	-	-	78–95	100	78–95
16 mm	70–88	-	77.18	67–87	-	67–87
12.5 mm	-	70–90	-	56–80	89.9	56–60
11.2 mm	-	-	63.28	-	-	-
9.5 mm	-	-	-	42–68	75.5	42–68
8.0 mm	50–66	-	54.96	-	-	-
5.66 mm	-	-	47.2	-	-	-
4.75 mm (no. 4)	-	40–65	38.4	29–57	55.7	29–57
2.0 mm (no. 10)	24–38	25–45	27.75	19–45	39.2	19–45
1.18 mm (no. 16)	-	-	-	14–34	-	14–34
1.0 mm (no. 18)	-	-	20.69	-	-	-
0.595 mm (no. 30)	11–21	-	-	10–25	18.8	10–25
0.42 mm (no. 40)	-	10–26	15.72	5–17	-	5–17
0.177 mm (no. 80)	8–6	6–18	10.41	3–10	-	-
0.149 mm (no. 100)	-	-	-	-	9.4	3–10
0.075 mm (no. 200)	-	3–8	-	-	6.5	1–7
0.063 mm (no. 230)	4.5–8	-	6.75	-	-	-

## 4. Structural Properties of Asphalt Layer

In terms of the physical properties of the asphalt layer, the main factors are the subgrade bearing capacity and the track settlement restriction. Traditionally, the total track settlement is superposed on the settlement in each layer [50].

Track settlement is relevant to the subgrade modulus, where the subgrade modulus is related to each layer’s thickness [51]. Hwang et al. [52]. suggested the following equation based on a nonlinear elastic multilayer system under the railway to calculate the dynamic modulus of a hot mix asphalt layer. This equation is based on the Asphalt Institute’s model:

(1) $$E\;=\;10^{5}\;+\;10^{\beta_{1}}$$

where β_1_ is a constant and is related to four other constants, which are β_2_, β_3_, β_4_, and β_5_.

In order to calculate β_1,_ the above four constants should be calculated. Then β_1_ will be plugged in into Equation (1) to determine the dynamic modulus of hot mix asphalt.

The following equations present how to calculate β_1_, β_2_, β_3_, β_4_, and β_5_:

(2) $$\beta_{1}\;=\;\beta_{3}\;+\;0.000005\;\beta_{2}\;-0.00189\;\beta_{2}f^{-1.1},$$

(3) $$\beta_{2}\;=\;\beta_{4}^{0.5}\;T^{\beta_{5}},$$

(4) $$\beta_{3}\;=\;0.553833\;+\;0.028829P_{200}f^{-0.1703}\;-\;0.03476V_{V}\;+\;0.070377\eta \;+\;0.931757f^{0.0277},$$

(5) $$\beta_{4}\;=\;0.483\;V_{b},$$

(6) $$\beta_{5}\;=\;1.3\;+\;0.49825\;\mathrm{Log}\;f.$$

The parameters presented in the above equations are as follows:

E is the dynamic modulus of the hot mix asphalt in pounds per square inch; β_1_, β_2_, β_3_, β_4_, and β_5_ are temporary constants; E is the dynamic modulus of elasticity (psi) for the HMA layer; T is the temperature (°F); P_200_ is the percent of aggregate passing sieve no. 200; *ƒ* is the load frequency; V_v_ is the air voids percentage; V_b_ is the binder content; η is the viscosity of the bitumen.

It is also possible to perform the dynamic stiffness tests on laboratory-made samples from the designed mix for the project at different temperatures and loading frequencies. The results will be used to construct the stiffness master curve of the asphalt, and then it is possible to determine the dynamic modulus at any desired temperature and loading frequency. In order to make the calculations easier, the developers of the Kentrack computer model have recommended using an empirical model, as suggested by the Mechanistic Empirical Pavement Design Guide (MPEDG), to calculate the dynamic modulus for hot mix asphalt. It is accepted that an asphalt layer is almost elastic at temperatures under 20 °C, with a dynamic modulus of elasticity between 5000 and 10,000 N/mm^2^. However, when the asphalt layer’s temperature exceeds 20 °C, plastic deformation is accumulated around the load contact area, which needs to be addressed and limited.

Based on the dynamic modulus of the asphalt and subgrade, the following equations, which were proposed by the Asphalt Institute [53], can be used to calculate the amount of allowable repetitive loading in an asphalt layer and over the subgrade:

(7) $$N_{a}\;=\;0.0795\;\varepsilon_{t}^{-3.291}\;E_{a}^{-0.854},$$

(8) $$N_{d}\;=\;4.837\;\times \;10^{-5}\;\sigma_{c}^{-3.734}\;E_{s}^{3.853},$$

where:

$N_{a}$ is the allowable number of repetitive loads in the asphalt layer;
$\varepsilon_{t}$ is the horizontal tensile strain under the asphalt layer;
$E_{a}$ is the asphalt dynamic modulus (psi);
$N_{d}$ is the allowable number of repetitive loads in the subgrade layer;
$\sigma_{c}$ is the compressive stress on top of the subgrade layer;
$E_{s}$ is the modulus of subgrade (psi).

Besides the asphalt dynamic modulus, another important parameter that needs to be calculated is the track modulus or stiffness. The traditional equation for calculating the track modulus is as follows [53,54]:

(9) $$u\;=\;\frac{1}{4}\sqrt[{3}]{\frac{P^{4}}{{\mathrm{EIW}}_{m}^{4}}},$$

where:

u is the subgrade elasticity (lb/in/in);
P is the amount of wheel load (lb);
E is the rail modulus of elasticity (psi);
I is the rail moment of inertia (in^4^);
$w_{m}$ is the deflection under the wheel (in).

This equation is common, especially in the USA, because it is easy to use, but the problem is that a special test setup is required for applying the load and measuring the deformation. To solve this problem, investigators have suggested simpler methods, such as the Plate Load Test (PLT), or have tried to use the results of the California Bearing Capacity (CBR) Test or the modulus of resilience (M_R_) in order to calculate the subgrade modulus of elasticity [55,56]. The amount of pressure produced over the asphalt layer under the sleeper is less than 0.3 N/mm^2^ for a typical passenger train, while it is between 0.7 and 0.9 N/mm^2^ for heavy trucks on highways [57,58].

The typical thickness for a bituminous layer on an Italian high-speed railway is between 12 to 14 cm, which lays over a densely compacted aggregate layer [59]. In this regard, rubber-modified asphalt can enhance the energy absorption properties of the sub-ballast layer for high-speed conditions [60]. However, in terms of energy absorption, both modified and non-modified bituminous sub-ballasts improve the track properties. In addition, careful consideration should be given to ensure that the track geometry and bearing capacity restrictions are suitable for high-speed railways [61]. In this regard, the asphalt layer should have the capability to decrease the magnitude of vertical stress to lower than 0.05 N/mm^2^ over the subgrade layer [62].

To determine the critical responses (vertical stress on the subgrade and horizontal strain under the asphalt layer), different computer programs can be used, from simple multilayer elastic models, up to more complicated ones that are also capable of considering the railway’s superstructure. Kentrack is a program that was developed to analyze railroad tracks, including the ones with an asphalt underlayment [63,64]. It is also possible to perform stiffness and fatigue tests directly on laboratory-produced asphalt samples to determine the main inputs for the computer model, to determine the critical responses, and to predict the lifespan (like the mechanistic-empirical approach), instead of using the abovementioned equations. Stiffness tests can be performed at different temperatures and loading frequencies to be able to construct the stiffness master curve of the asphaltic material, as well as to consider the effect of train speeds, or the temperature changes on the stiffness of the asphaltic layer.

## 5. Asphalt Performance and Constructability

Considering that an asphalt layer does not require curing after being laid on the track bed, it is a suitable material for ballastless track applications, specifically when sleepers are laid directly on it. A survey on construction progress, in meters per day of construction, for various ballastless tracks showed Rheda 2000 has the highest performance because it is totally mechanized. Other than that, track systems with asphalt layers are the systems with high productivity compared to other concrete slab systems [24]. In the case of asphalt pavement, it cools down in two to three hours, reaching a minimum compressive pressure resistance of 12 N/mm^2^, while it takes three to seven days for a concrete slab to reach the same amount. For asphalt, in the case of hot mixes, the main point is related to logistics and transporting the mix from the production facility to the laydown place. Recent developments in the area of mobile mixing plants have made this issue less critical.

In order to evaluate the performance of an asphalt layer, several [65,66] performed a numerical analysis on an asphalt layer under a high-speed railway and concluded that the optimal location of the asphalt layer is under the ballast and over the subgrade, which will lead to a higher bearing capacity in the track bed. Moreover, in order to decrease construction costs, a specific asphalt mortar pouring method with a long pipe was recommended for a line located in a snowy region of Japan [67]. In terms of construction cost, a research study on Spain’s high-speed railway track revealed that in many cases, the construction costs of tracks with a bituminous sub-ballast were similar to tracks with an all granular sub-ballast [68].

## 6. Review of Case Studies

This section focuses on a review of investigations performed on asphalt pavement and mortar under a railway track. Considering that researchers have shown interest in both traditional and modified asphalt, the findings for each are presented in one subsection. The last subsection also provides the results of investigations on asphalt concrete under a railway track.

### 6.1. Ballasted Railway Tracks with Asphalt

Studies have revealed a positive influence, as well as benefits, for using asphalt as a track layer. In an experimental study [20], sections of 10 to 30 cm of an asphalt layer were installed under various railway tracks. Based on the results, after seven years, the sections having asphalt underlayment required less geometrical realigning and re-ballasting compared to a ballasted track. In addition, the subgrade under the asphalt layer was found to remain uniform. On-site investigations of a trial railway section with Y-shape sleepers over two layers of asphalt pavement with a 15 cm thickness presented a higher lateral resistance and lower vibration rate, which means a longer track lifecycle [69].

Moreover, asphalt creates a waterproofing layer, with better separation between the ballast and subgrade layers, which helps to eliminate the negative impact of a mud pumping subgrade and ballast fouling [38]. A layer of thick hot mix asphalt increases the overall modulus of the track packet compared to a ballasted one. The higher track modulus leads to lower strain under an asphalt layer. Therefore, it is necessary to find and recommend an optimal thickness for an asphalt layer. In this regard, a theoretical analysis suggested an asphalt layer with a thickness of 12 to 14 cm to be a sufficient alternative for a 30 cm traditional sub-ballast layer [70].

A bituminous layer is an appropriate alternative to traditional granular materials and helps to improve track performance by decreasing the track settlement and enhancing the track bearing capacity [53], track stiffness [71,72,73,74], and stability and fatigue resistance [75,76,77], as well as reducing the track’s vertical acceleration [78], and its overall thickness, especially in tunnels [79]. Furthermore, in a trial section under the Train à Grande Vitesse (TGV) high-speed railway, French experts were able to decrease the total thickness of the track by 21 cm by replacing a 35 cm base layer with a 14 cm asphalt layer between the ballast and formation layers. The asphalt modulus used in this section was over 11,000 MPa [3].

A transition zone, which is the section between a ballasted track and a slab track, or more specifically, the section on both sides of a concrete bridge slab in a ballasted track, is required to be designed and installed [80], One of the secondary applications of the asphalt is that it is an ideal material that can be applied in transition zones to restrict permanent settlement [81,82], increase the track modulus, and overcome the stiffness differences [83]. between the ballast and bridge’s or tunnel’s concrete deck. A layer of 20 cm of asphalt underlayment with a length of over 60 m [84,85] is one recommended method [86] for a transition zone, while a 30 cm asphalt underlayment behaves similar to 20 cm of concrete layer over the subgrade. For instance, the design method used for renovating bridge approach sections by the CSX railroad (in the USA) included a 12.2 m long asphalt underlayment with a 15 cm thickness [87].

More recent long-term monitoring projects show asphalt’s significant influence in distributing the load and reducing the amount of pressure on a track’s bed [88], thereby decreasing its settlement [89]. Asphalt underlayment presents benefits for both high-speed and freight railway [90], as well as in cases of heavy-haul railways [91]. In contrast, a two-year experimental study on 105 km of a high-speed ballasted track with asphalt underlayment in France displayed a reduction in the acceleration level under the ballast layer, but no significant improvement in track settlement for a track with an asphalt underlayment in comparison to a track with conventional granular material. Based on this study, the asphalt underlayment section presented better drainage properties and embankment protection. Another point about using asphalt underlayment is that the ballast layer provides a kind of protection against severe temperature changes [92,93]. In addition, investigations are ongoing by French railway authorities to develop an asphaltic layer as an alternative for freight traffic, especially in tunnels, to increase the tunnel gauge by decreasing the overall thickness of the track [94].

Modifying the ballast with a bitumen emulsion is another method that some experts have suggested for enhancing ballast properties. Bitumen brings more cohesion into the mix, enhancing its shear resistance parameters, and consequently, improving its mechanical and response properties regarding cyclic loading. The ballast gradation, along with the emulsion’s type and dosage, are among the important factors that have an influence on the outcome [95]. Bitumen emulsion also decreases permanent deformation, as well as the deformation rate in the ballast layer [89], along with particle degradation, by preventing particle movement and friction [96]. Emulsion increases a ballast’s durability, especially for early applied cases [97]. An emulsion-modified ballast requires a different maintenance strategy because the modification can lead to a more sustainable ballast, and therefore, to an increase in the maintenance intervals [98,99]. This method of stabilizing the ballast particles was first introduced by a team of researchers at Heriot-Watt University in 2004 [100,101].

### 6.2. Ballastless Railway Track with Asphalt

In an analytical research study, the behavior of various railway substructures with three different HMA applications was investigated [21]. The three asphalt applications included: system 1—a concrete slab track, system 2—a concrete slab over an asphalt pavement, and system 3—adding a reinforced concrete layer between the asphalt layer and the concrete slab. The results demonstrated a similarity between the dynamic responses of the slab track (system 1) and the asphalt layer under reinforced concrete (system 3). Moreover, the asphalt layer improved the resilience performance of the track substructure, as well as the stress distribution. In addition, the various concepts of dynamic loading [102], and its influence on the behavior of the railway track over an asphalt slab bed was presented by some investigators [103]. 3D finite element model and laboratory tests were proposed by other investigators to analyze the dynamic behavior of the track bed [104,105]. It has to be mentioned that, despite having some differences, the in situ experiments usually validated the acceptance of the dynamic and numerical modeling [106,107]. Based on a finite element model, the use of an asphalt layer improves the sleeper support [108]. Dynamic modes were also used to analyze the influence of temperature on an asphalt layer in a slab track, as well as the waterproofing properties of the bituminous layer [109,110]. Several case studies and models in China have recommended self-compacted and mastic asphalt due to its effectiveness at enhancing the waterproofing properties of the asphalt layer [111,112].

A full-scale experiment on a Korean railway presented the benefits of using asphalt for railway systems. In this study, the optimal thickness for an asphalt layer was suggested to be 30 cm after applying static loading on track beds with three different thicknesses. The authors also discovered that the amount of transverse strain under the asphalt layer was less than 100 microstrain, which means that the asphalt layer had the capability to withstand the train’s load [113]. Another experimental investigation by this team showed 3 mm of settlement for a track made of a 35 cm asphalt layer under cyclic loading [114]. Asphalt also increases the permanent deformation resistance of the track and remarkably improves the durability and service life of the track [115], while the roadbed pressure and strain underneath the asphalt layer remain in the acceptable range in the long-term [116].

### 6.3. Railway Tracks with Modified Asphalt Mixtures

The performance of asphalt pavement modified with various additives, such as rubber and polymer under the track bed, was considered in some studies. An investigation about using rubber-modified asphalt illustrated that dry asphalt rubber concrete improves the sub-ballast fatigue resistance [117,118]. In particular, it improves the track elasticity and its effectiveness in damping vibrations [119]. Furthermore, it has been discovered that asphalt modified with 10 to 20% rubber leads to an improvement in the shear modulus and damping ratio, though both factors are significantly related to temperature [120]. In addition, for asphalt modified with 20% rubber, the penetration resistance is higher compared to traditional, non-modified asphalt [121].

Polymer modified asphalt, on the other hand, increases the elastic recovery of the binder, as well as the viscosity and strength of the asphalt [122]. Moreover, in a comparison between the behaviors of Styrene-Butadiene-Styrene (SBS)-modified asphalt with rubber-modified asphalt under a railway [35], both presented higher elastic and viscoelastic behaviors under a uniaxial creep test, while the SBS-modified one, demonstrated a higher stiffness. With a 5.5% optimum binder content, SBS-polymer-modified asphalt displayed the highest resistance to fatigue deterioration and rutting [41].

### 6.4. Railway Tracks with Asphalt Mortar

In an investigation on slab tracks used on a Chinese high-speed railway, the team proposed using reactive powder concrete materials to decrease the cracks at the slab, asphalt mortar, and roadbed [123]. Another study on asphalt mortar revealed that the flexural strength of mortar decreases with the increase of temperature [124]. Asphalt mortar is a sensitive material when exposed to temperature and constant pressure; it deteriorates more as the pressure and temperature increase [125]. Furthermore, in order to design asphalt mortar with a slab track, special consideration needs to be given to predict the mortar’s deterioration and shock-absorbing properties of bitumen emulsion and fine aggregate admixture [126].

An increase in asphalt mortar thickness minimizes the amount of maximum tensile strain and the magnitude of track deformation under cyclic loading. The recommended thickness of asphalt mortar is between 10 and 50 mm, with a modulus of elasticity between 7000 and 10,000 N/mm^2^. Higher asphalt mortar stiffness leads to a reduction in slab track bending stresses, and consequently, track deformation [29]. Based on dynamic analysis models proposed by different researchers, for a moving wheel load, asphalt slab tracks create firm support for sleepers while decreasing the noise rate under dynamic loading [127,128,129,130,131]. One important factor when analyzing the asphalt mortar under the railway track is the temperature and its influence on the behavior of mortar. Higher temperatures lead to a decrease in the mortar’s dynamic modulus [132]. In this regard, some experts have suggested more in-depth analyses to reveal the correlation between the mortar’s dynamic properties and various temperatures [133]. Temperature and asphalt/cement volume ratio are two parameters having influence on the mortar’s compressive strength [134]. Moreover, other researchers investigated the bonding between mortar and slab, and because this bond is generally not strong, some suggested adding polymer latex to improve the bonding strength of the mortar, as well as a quick-hardening admixture. In addition, improving the mortar strength provides reasonable support to the joints [135,136,137,138,139].

## 7. Asphalt Application in Urban Railway Tracks

**Figure 5:**
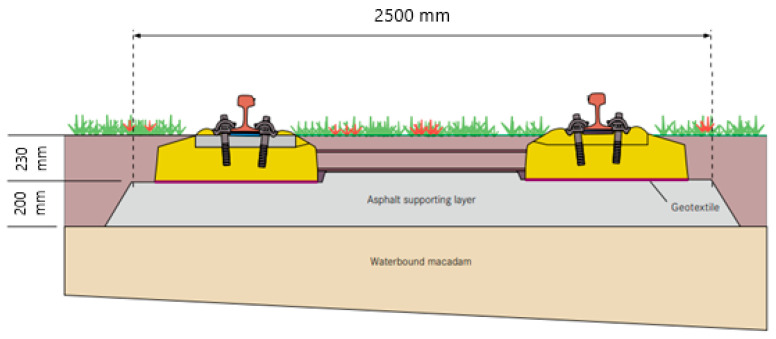
Schematic of a light rail with an asphalt track bed (With permission from RAIL.ONE) [142].

Asphalt has also has been used extensively in the construction of urban railway tracks. The most well-known samples for this type of track are embedded track and tram system tracks. Typically, urban trains have lighter weights, and for an urban track, the axle load is less than for a regular railway. In these systems, asphalt is used for structural purposes or filling, as well as a combination of both. Figure 5 presents a schematic of a light rail with an asphalt track bed.

One method of using asphalt as a structural element in urban tram tracks is the Nikex (webless) system. This system, which includes three layers of asphalt under a concrete slab track, was used in the Netherlands in the early 1980s to build tram tracks [140]. The propagation of waves over the rail profile was a limiting factor behind why this system was not successful [141].

Another well-developed urban railway system is embedded rail. The type of asphalt used in an embedded track is different than regular asphalt. In this system, a 20 cm layer, which is called surface asphalt concrete (SAC), is laid over unbound crushed masonry to create a base for embedding the asphalt layer. The air void content in this type of asphalt is relatively high; in the range of 15 to 20%, a kind of cement-based mortar mix is used to fill these voids. This type of asphalt is considered to have higher strength and stiffness, as well as better bearing capacity against settlement under heavy loading [143]. However, the properties of this type of asphalt are considerably related to temperature [144].

## 8. Summary and Conclusions

In this paper, different aspects of the application of asphaltic and bituminous materials under a railway track were investigated. Investigations about all types of asphalt applications under a railway track, including underlayment, overlayment, slab track, and asphalt mortar, as well as applying different modified asphalts, were reviewed. Table 7 presents a summary of various asphalt and bituminous materials that are common or have been studied in different countries.

Moreover, the results of this investigation are summarized as follows:

1. The use of asphalt underlayment with a traditional ballasted track leads to an increase in the track stiffness and a decrease in its overall settlement. This is an important parameter that has a direct influence on track maintenance costs. Moreover, ballasted tracks with asphalt underlayment will have lower maintenance in comparison to those with a traditional granular sub-ballast.
2. The amount of stress and strain under a railway track with asphalt underlayment is mainly related to the layer thickness, but in general, replacing granular materials with asphalt pavement can decrease the overall height of the ballasted railway track. In tunnels, especially, this parameter is a dominant factor, which means a reduction in the overall tunnel height and cross-sectional area. Asphalt also has waterproofing properties, which protect the subgrade from moisture-related damage.
3. In addition, asphalt is a good alternative to a concrete slab when constructing ballastless railway tracks for high-speed and urban systems. It provides a good level of stiffness balance in the whole track system, which is crucial for its overall stability (acceptable level of settlement), and simultaneously, its response to the dynamic loads of high-speed trains. In France and Italy, specifications and standards have been established to cover the use of the asphalt slab track under high-speed railways. Countries like Spain and Austria have already used asphalt when constructing high-speed railways. In Germany, different methods, such as Gentrac and ATD, have been introduced, which are based on using asphalt as a bearing capacity layer for ballastless tracks. Moreover, thanks to the advances in construction machines, constructing a ballastless track with asphalt pavement is nowadays precise, and in most cases, faster than concrete slabs.
4. Other countries, such as Japan and China, are currently using asphalt mortar between the concrete slab and concrete bed as part of their design systems for slab tracks. In these countries, numerous studies have been performed on this system, which presents the positive influence of an asphalt mortar layer on a slab track.
5. Additionally, other studies on modified asphalt present the feasibility of using a modified bitumen binder and asphalt mixture in railway tracks. In this regard, both polymer and rubber-modified asphalts add significant benefits to the performance of track systems.

## Figures and Tables

**Table 1:** Lengths and thicknesses of railway tracks with bituminous layers in various countries.

Country	Germany [7]	Italy [8]	France [9]	Morocco [9]	Spain [10]	China [11]	Japan [11]	USA [12]
Length (km)	82	1200	300	200	300	2860	2200	322
Asphalt/asphalt mortar thickness (cm) [13]	20–30	12	14	14	12–14	3–5	5	12–30

**Table 2:** Properties of a typical track with asphalt underlayment used in the USA [20,21].

Layer	Thickness (cm)	Minimum Modulus (MPa)	Poisson’s Ratio	Unit Weight (kg/m^3^)
Ballast	20–30	50	0.35	1800
Asphalt	15–30	4200	0.45	2400
Sub-ballast	10	25	0.35	1800
Subgrade	-	85	0.4	2200

**Table 3:** Specifications of asphalt bases in ballastless systems [24].

Method	Asphalt Thickness (mm)	Superstructure Overall Thickness (mm)	Weight (ton/m)
ATD	300	1021	2.7
SATO	300	909	2.2
FFYS	300	909	2.2
Getrac	300	1021	2.7
Walter	300	929	2.3

ATD: asphalt track bed directly laid on the sleeper, SATO, FFYS.

**Table 4:** Marshall mix design acceptance rates [34].

Mix Criteria	Light Traffic (<104 ESALs)		Medium Traffic (104–106 ESALs)		Heavy Traffic (>106 ESALs)	
	Min.	Max.	Min.	Max.	Min.	Max.
Compaction (blows per each side)	35		50		75	
Minimum stability (minimum)	2224 N		3336 N		6672 N	
Flow (0.25 mm)	8	20	8	18	8	16
Air voids (%)	3	5	3	5	3	5

ESALs: equivalent single axle loads.

**Table 5:** Recommended properties of asphalt for railways.

Study Reference	Compaction (Blow Number)	Stability (N)	Flow	V_a_ (%)	VFA (%)	VMA (%)	Binder Content (%)	Density (kg/m^3^)
USA [38]	50	3375	3.8–6.4	1–3	80–90		6.5–7.4	2240
Italy [39]	102–291	10,000	-	2–4	75.4–82.4	12.4–18.6	4–7	2500
Korea [35]	-	-	-	3	-	-	4.4	2496
Iran [40]	50	9400	2.5	8.5	-	-	-	2216
Korea [41]	-	>4900	20–40	3	70–80	>13	5.2–5.5	2353
Spain [42]	50–75	11,850	3.9	2.8	-	-	4.25	2650
France [43]	-	-	-	3–4	-	-	4.8	-
China [44]	-	8000	-	1–3	89–91	12.6–16	4.5–5.8	2525

V_a_: air void ratio, VFA: voids filled with asphalt, VMA: voids in mineral aggregates.

**Table 7:** Various applications of asphalt and bituminous materials under railways.

Country	Type	Common Thickness (cm)	Speed
USA	Asphalt underlayment	15–30	Regular
Germany	Slab track	20–30	High speed
France	Asphalt underlayment	14	High speed
Italy	Slab track	12	High speed
Japan	Bitumenus mortar	5	High speed
China	Bituminous mortar	5	High speed
